# Beyond dose escalation in glioblastoma: Lessons learned from INTRAGO-II and the case for targeting tumor biology

**DOI:** 10.1093/neuonc/noag166

**Published:** 2026-09-19

**Authors:** Julian Philipp Layer, Matthias Schneider, Michael Hölzel, Ulrich Herrlinger, Frank Winkler

**Affiliations:** Department of Radiation Oncology, University Hospital Bonn, University of Bonn, Bonn, Germany; Institute of Experimental Oncology, University Hospital Bonn, University of Bonn, Bonn, Germany; Department of Neurosurgery, University Hospital Bonn, University of Bonn, Bonn, Germany; Brain Tumor Translational Research Group, University Hospital Bonn, University of Bonn, Bonn, Germany; Institute of Experimental Oncology, University Hospital Bonn, University of Bonn, Bonn, Germany; Brain Tumor Translational Research Group, University Hospital Bonn, University of Bonn, Bonn, Germany; Department of Neuro-Oncology, University Hospital Bonn, University of Bonn, Bonn, Germany; Neurology Clinic, National Center for Tumor Diseases & European Center for Neurooncology (EZN), University Hospital Heidelberg, Heidelberg University, Heidelberg, Germany; Clinical Cooperation Unit Neurooncology, German Cancer Consortium (DKTK), German Cancer Research Center (DKFZ), Heidelberg, Germany

The results of INTRAGO-II, the largest randomized trial to date testing focal radiation dose escalation in newly diagnosed glioblastoma, have been recently published in *Lancet Oncology*.^1^ The addition of intraoperative radiotherapy, delivering an additional ultrahigh dose of 30 Gy to the resection cavity, did not improve progression-free survival (PFS), overall survival (OS), or, surprisingly, even local tumor control. While the trial result is utterly disappointing, its implications for the field of neuro-radiation oncology are profoundly important and require further action.

INTRAGO-II provides definitive level-I evidence against focal dose escalation in glioblastoma, closing the case on a decades-long series of ambiguous results.^2^ Since 60 Gy has been established as the effective dose ceiling for external-beam radiotherapy several decades ago, multiple strategies including both focal and large-field, brachytherapy and external-beam radiotherapy concepts have been tested and ultimately failed. INTRAGO-II now extends this pattern to a radiation modality offering the steepest achievable dose gradient and the unique advantage of immediate post-resection delivery. Moreover, the multicentric trial with adequately sized cohorts was conducted in a modern clinical trial environment with thorough central radiology review, thus preventing wrong endpoint assignments. Taken together, the evidence against dose escalation in glioblastoma is now comprehensive and it may be time for the scientific community to accept this fact and intensify efforts questioning the *why*.

Several observations from INTRAGO-II suggest that the barrier to improved local control is fundamentally biological, not physical. Approximately 70% of recurrences in both arms occurred within 1 cm of the resection cavity, indicating that intraoperative radiotherapy boosting did not alter the dominant recurrence pattern despite substantial dose delivery. Most strikingly, even patients with measurable residual tumor, where the delivered dose was unequivocally within the therapeutic dose range, showed no detectable benefit. This challenges the central assumption underlying all dose escalation strategies that glioblastoma cells are amenable to dose-dependent cytotoxicity.

Multiple biological mechanisms may underlie this finding, including potentially insurmountable intrinsic radioresistance of residual glioblastoma cells. The discovery of radioresistant tumor microtube-connected communicating and resilient tumor cell networks^3^ and the increase of radiation efficacy by pharmacological and genetic network disconnection strategies^4^ provide a new framework how to understand and to therapeutically overcome multicellular mechanisms of treatment resistance. In addition, radiotherapy-induced pericavitary microenvironmental changes including increased vascular permeability, hypoxia, cell senescence, cell death and chronic inflammation may induce pathways that paradoxically support tumor survival, revascularization^5^ and depletion of immune-competent cells in the irradiated area.^6^ These considerations collectively argue for a paradigm shift from physical dose intensification toward biologically informed, more tailored systemic treatment strategies.

Importantly, the consistent failure of dose escalation, now culminating in a definitive phase 3 trial, should not be interpreted as a failure of radiotherapy. Rather, it is a warning sign that the biological determinants of glioblastoma radioresistance require therapeutic combination strategies that modulate detrimental cancer-intrinsic and post-radiogenic processes, including adequately addressing tumor networks outside of the surgery and radiation fields. Future trials should prioritize such network disconnecting or microenvironment-targeted combinatorial approaches over further physical dose intensification in a disease where this concept has been comprehensively tested and disproven. It is about time to move on.
